# Supplementary material for: A fungal core effector exploits the OsPUX8B.2–OsCDC48-6 module to suppress plant immunity
Source: Nat Commun. 2024 Mar 22;15:2559. doi: 10.1038/s41467-024-46903-7 (PMC10959940; doi:10.1038/s41467-024-46903-7)
Supplement: Supplementary file 1 — Supplementary information [file 41467_2024_46903_MOESM1_ESM.pdf]

Supplementary information

A fungal core effector exploits the OsPUX8B.2–OsCDC48-6 module to suppress plant immunity

Xuetao Shi[1,2], Xin Xie[1], Yuanwen Guo[1], Junqi Zhang[1], Ziwen Gong[1,2], Kai Zhang[1], Jie Mei[1,2], Xinyao Xia[1], Haoxue Xia[1], Na Ning[1], Yutao Xiao[2], Qing Yang[1], Guo-Liang Wang[3] and Wende Liu[1, ✉]

[1] State Key Laboratory for Biology of Plant Diseases and Insect Pests, Institute of Plant Protection, Chinese Academy of Agricultural Sciences, Beijing 100193, China

[2]Shenzhen Branch, Guangdong Laboratory of Lingnan Modern Agriculture, Key Laboratory of Gene Editing Technologies (Hainan), Ministry of Agriculture and Rural Affairs, Agricultural Genomics Institute at Shenzhen, Chinese Academy of Agricultural Sciences, Shenzhen 518000, China

[3] Department of Plant Pathology, The Ohio State University, Columbus, OH 43210, USA

✉ Correspondence: Wende Liu (liuwende@caas.cn)

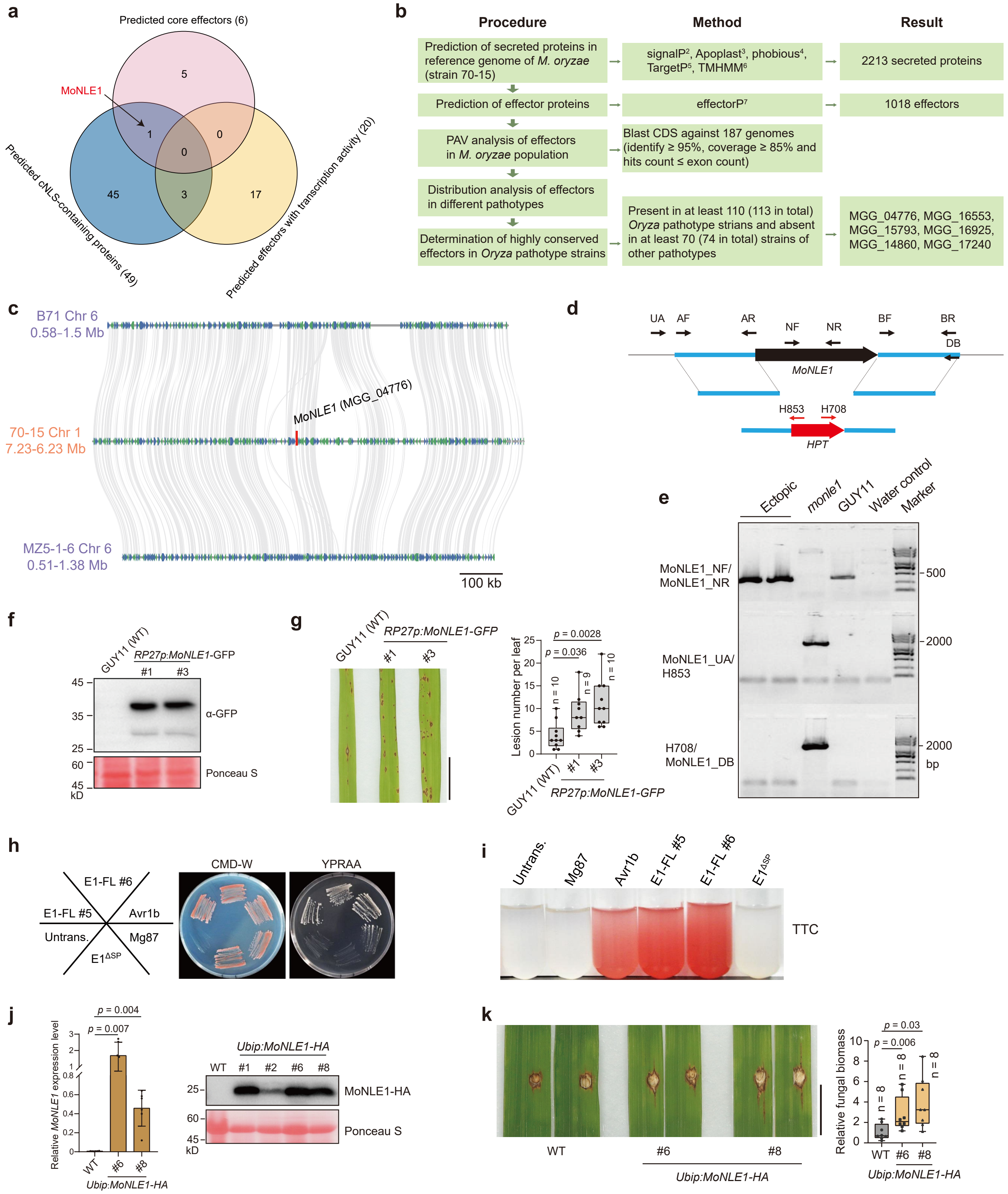
a
Predicted core effectors (6)
MoNLE1
5
1
0
0
45
3
17
Predicted cNLS-containing proteins (49)
Predicted effectors with transcription activity (20)
b
Procedure
Method
Result
Prediction of secreted proteins in reference genome of M. oryzae (strain 70-15)
signalP2, Apoplast3, phobious4, TargetP5, TMHMM6
2213 secreted proteins
Prediction of effector proteins
effectorP7
1018 effectors
PAV analysis of effectors in M. oryzae population
Blast CDS against 187 genomes (identify ≥ 95%, coverage ≥ 85% and hits count ≤ exon count)
Distribution analysis of effectors in different pathotypes
Determination of highly conserved effectors in Oryza pathotype strains
Present in at least 110 (113 in total) Oryza pathotype strians and absent in at least 70 (74 in total) strains of other pathotypes
MGG_04776, MGG_16553, MGG_15793, MGG_16925, MGG_14860, MGG_17240
c
B71 Chr 6
0.58-1.5 Mb
MoNLE1 (MGG_04776)
70-15 Chr 1
7.23-6.23 Mb
MZ5-1-6 Chr 6
0.51-1.38 Mb
100 kb
d
UA
AF
AR
NF
NR
BF
BR
DB
MoNLE1
H853
H708
HPT
e
Ectopic
monle1
GUY11
Water control
Marker
MoNLE1_NF/
MoNLE1_NR
MoNLE1_UA/
H853
H708/
MoNLE1_DB
500
2000
2000
bp
f
GUY11 (WT)
RP27p:MoNLE1-GFP
#1
#3
45
35
25
60
45
kD
α-GFP
Ponceau S
g
GUY11 (WT)
RP27p:MoNLE1-GFP
#1
#3
p = 0.0028
p = 0.036
Lesion number per leaf
n = 10
n = 9
n = 10
RP27p:MoNLE1-GFP
h
E1-FL #6
E1-FL #5
Avr1b
Untrans.
Mg87
E1ΔSP
CMD-W
YPRAA
i
Untrans.
Mg87
Avr1b
E1-FL #5
E1-FL #6
E1ΔSP
TTC
j
p = 0.004
p = 0.007
Relative MoNLE1 expression level
WT
#6
#8
Ubip:MoNLE1-HA
Ubip:MoNLE1-HA
WT
#1
#2
#6
#8
25
60
45
kD
MoNLE1-HA
Ponceau S
k
WT
#6
#8
Ubip:MoNLE1-HA
p = 0.03
p = 0.006
Relative fungal biomass
n = 8
n = 8
n = 8
WT
#6
#8
Ubip:MoNLE1-HA

Supplementary Fig. 1 Identification of MoNLE1 as a putative virulence effector. a, Venn diagram showing the extent of overlap between predicted cNLS-containing proteins identified in this study, previously predicted effectors with transcriptional activity[1], and predicted putative core effectors in the *Oryza* pathotypes of *M. oryzae*. The number of proteins for each dataset is shown in parentheses. **b,** Workflow for the identification of putative core effectors in the *Oryza* pathotypes of *M. oryzae*. PAV, presence/absence variation. **c,** Synteny plot comparing the genomic structure of rice blast isolate 70-15 and that of its homologs in *Triticum* and *Eleusine* within a 1-Mb region around the *MoNLE1* locus. The plot identifies complex structural variations near *MoNLE1* in the genomic architecture of these isolates. **d,** Diagram of the strategy used to generate the *monle1* mutant. Black arrows and blue lines indicate the primers and homologous arms, respectively, used to generate and identify *monle1*. *HPT*, *hygromycin phosphotransferase* gene. Primer sequences can be found in Supplementary Data 5. **e,** PCR identification of *monle1*. Primer positions are shown in (**d**). Ectopic transformants display off-target integration of the *HPT* gene in the fungal genome and were used as negative controls in the pathogenesis test. The blast strain GUY11 was used as the wild-type (WT) control. **f,** Validation of *RP27p:MoNLE1-GFP* transformants using immunoblotting. Ponceau S was used as loading control. **g,** Pathogenicity test of *RP27p:MoNLE1-GFP* transformants on rice (cultivar CO39). Data were recorded at 7 d post inoculation (dpi) and are shown as boxplots displaying the maximum and minimum, first and third quantiles, and the median. *n* = Number of independent biological samples. Scale bar, 1 cm. **h, i,** Yeast trap assay showing the secretion of MoNLE1 from yeast cells. Untransformed yeast cells were used as a transformation control; Mg87 and MoNLE1 with the signal peptide deleted ($E1^{\Delta SP}$) were used as negative controls; Avr1b was used as a positive control. Two yeast transformants producing full-length MoNLE1 (E1-FL#5 and E1-FL#6) were used to check for protein secretion. **j,** Validation of *Ubip:MoNLE1-HA* heterologous expressing plants in the Nipponbare (NPB) background by RT-qPCR (left panel) and immunoblotting (right panel). Data are means ± s.d. from six independent biological replicates for RT-qPCR analysis. Ponceau S staining was used as a loading control in the immunoblotting assay. **k,** Rice plants expressing *Ubip:MoNLE1-HA* show enhanced susceptibility to blast strain GUY11. Data were recorded at 15 dpi and quantified by relative fungal biomass. In (**g, j** and **k**), statistical analysis was performed with one-way ANOVA followed by Tukey's test and the adjusted *p* values were shown in figures. Immunoblot results shown in (**f**, **j**) are representatives of two independent experiments with similar results.

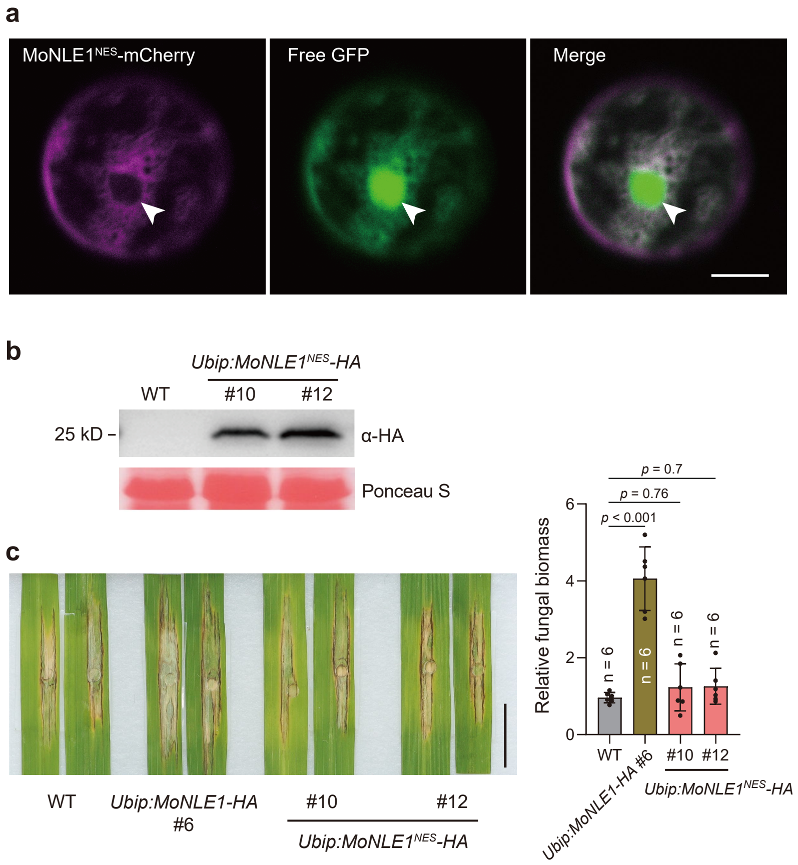

Supplementary Fig. 2 The NLS is required for MoNLE1 virulence. a, Subcellular localization analysis of MoNLE1^{NES} in rice protoplasts transfected with the indicated constructs. Arrowheads indicate the rice nucleus. **b,** Immunoblot determination of MoNLE1^{NES}-HA in rice plants expressing *Ubip:MoNLE1^{NES}-HA* with an anti-HA antibody. Data are representative of two independent experiments with similar results. **c,** Phenotype of *Ubip:MoNLE1^{NES}-HA* plants following infection with blast isolate RB22. Data were recorded at 12 d post inoculation (dpi) and quantified by relative fungal biomass. The adjusted *p* values were determined by one-way ANOVA followed by Tukey's test. Scale bar, 1 cm.

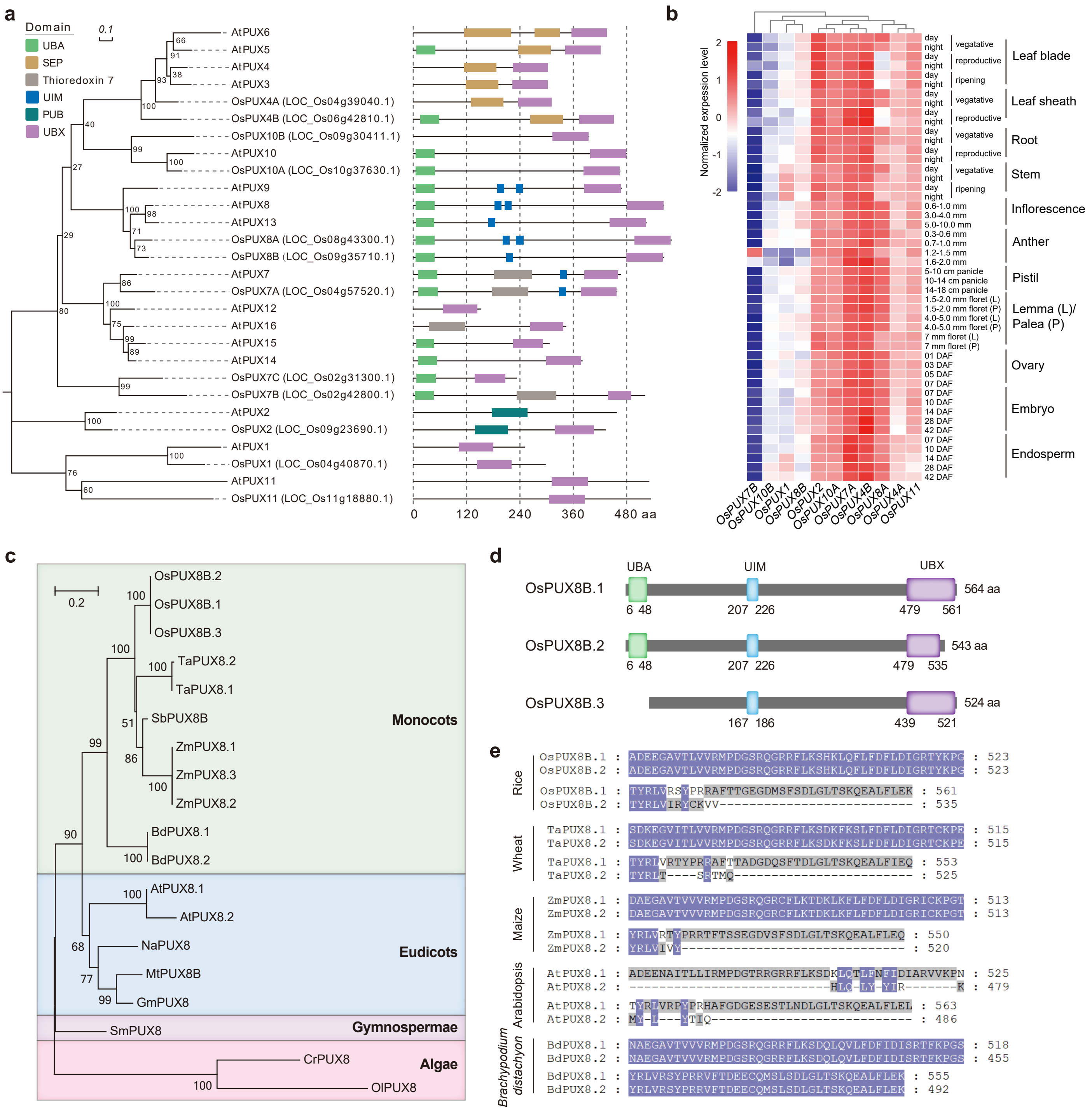
a
Domain
UBA
SEP
Thioredoxin 7
UIM
PUB
UBX
0.1
AtPUX6
AtPUX5
AtPUX4
AtPUX3
OsPUX4A (LOC_Os04g39040.1)
OsPUX4B (LOC_Os06g42810.1)
OsPUX10B (LOC_Os09g30411.1)
AtPUX10
OsPUX10A (LOC_Os10g37630.1)
AtPUX9
AtPUX8
AtPUX13
OsPUX8A (LOC_Os08g43300.1)
OsPUX8B (LOC_Os09g35710.1)
AtPUX7
OsPUX7A (LOC_Os04g57520.1)
AtPUX12
AtPUX16
AtPUX15
AtPUX14
OsPUX7C (LOC_Os02g31300.1)
OsPUX7B (LOC_Os02g42800.1)
AtPUX2
OsPUX2 (LOC_Os09g23690.1)
AtPUX1
OsPUX1 (LOC_Os04g40870.1)
AtPUX11
OsPUX11 (LOC_Os11g18880.1)
0
120
240
360
480 aa
b
Normalized expression level
2
1
0
-1
-2
Leaf blade
Leaf sheath
Root
Stem
Inflorescence
Anther
Pistil
Lemma (L)/
Palea (P)
Ovary
Embryo
Endosperm
vegatative
reproductive
ripening
day
night
0.6-1.0 mm
3.0-4.0 mm
5.0-10.0 mm
0.3-0.6 mm
0.7-1.0 mm
1.2-1.5 mm
1.6-2.0 mm
5-10 cm panicle
10-14 cm panicle
14-18 cm panicle
1.5-2.0 mm floret (L)
1.5-2.0 mm floret (P)
4.0-5.0 mm floret (L)
4.0-5.0 mm floret (P)
7 mm floret (L)
7 mm floret (P)
01 DAF
03 DAF
05 DAF
07 DAF
10 DAF
14 DAF
28 DAF
42 DAF
OsPUX7B
OsPUX10B
OsPUX1
OsPUX8B
OsPUX2
OsPUX10A
OsPUX7A
OsPUX4B
OsPUX8A
OsPUX4A
OsPUX11
c
0.2
OsPUX8B.2
OsPUX8B.1
OsPUX8B.3
TaPUX8.2
TaPUX8.1
SbPUX8B
ZmPUX8.1
ZmPUX8.3
ZmPUX8.2
BdPUX8.1
BdPUX8.2
AtPUX8.1
AtPUX8.2
NaPUX8
MtPUX8B
GmPUX8
SmPUX8
CrPUX8
OlPUX8
Monocots
Eudicots
Gymnospermae
Algae
d
UBA
UIM
UBX
OsPUX8B.1
564 aa
6 48
207 226
479 561
OsPUX8B.2
543 aa
6 48
207 226
479 535
OsPUX8B.3
524 aa
167 186
439 521
e
Rice
OsPUX8B.1 : ADEEGAVTLVVRMPDGSRQGRRFLKSHKLQFLFDFLDIGRTYKPG : 523
OsPUX8B.2 : ADEEGAVTLVVRMPDGSRQGRRFLKSHKLQFLFDFLDIGRTYKPG : 523
OsPUX8B.1 : TYRLVRSYPRRAFTTGEGDMSFSDLGLTSKQEALFLEK : 561
OsPUX8B.2 : TYRLVIRYCKVV-------------------------- : 535
Wheat
TaPUX8.1 : SDKEGVITLVVRMPDGSRQGRRFLKSDKFKSLFDFLDIGRTCKPE : 515
TaPUX8.2 : SDKEGVITLVVRMPDGSRQGRRFLKSDKFKSLFDFLDIGRTCKPE : 515
TaPUX8.1 : TYRLVRTYPRRAFTTADGDQSFTDLGLTSKQEALFIEQ : 553
TaPUX8.2 : TYRLT----SRTMQ------------------------ : 525
Maize
ZmPUX8.1 : DAEGAVTVVVRMPDGSRQGRCFLKTDKLKFLFDFLDIGRICKPGT : 513
ZmPUX8.2 : DAEGAVTVVVRMPDGSRQGRCFLKTDKLKFLFDFLDIGRICKPGT : 513
ZmPUX8.1 : YRLVRTYPRRTFTSSEGDVSFSDLGLTSKQEALFLEQ : 550
ZmPUX8.2 : YRLVIVY------------------------------ : 520
Arabidopsis
AtPUX8.1 : ADEENAITLLIRMPDGTRRGRRFLKSDKLQTLFNFIDIARVVKPN : 525
AtPUX8.2 : ---------------------------HLQ-LY-YIR-------K : 479
AtPUX8.1 : TYRLVRPYPRHAFGDGESESTLNDLGLTSKQEALFLEL : 563
AtPUX8.2 : MY-L---YTIQ--------------------------- : 486
Brachypodium distachyon
BdPUX8.1 : NAEGAVTVVVRMPDGSRQGRRFLKSDQLQVLFDFIDISRTFKPGS : 518
BdPUX8.2 : NAEGAVTVVVRMPDGSRQGRRFLKSDQLQVLFDFIDISRTFKPGS : 455
BdPUX8.1 : YRLVRSYPRRVFTDEECQMSLSDLGLTSKQEALFLEK : 555
BdPUX8.2 : YRLVRSYPRRVFTDEECQMSLSDLGLTSKQEALFLEK : 492

Supplementary Fig. 3 Phylogenetic analysis, domain organization, and tissue-specific expression patterns of PUX family members. a, Phylogenetic analysis of PUX family members in rice and Arabidopsis. The phylogenetic tree was reconstructed using the neighbor-joining method in MEGA 7 with 12 rice and 16 Arabidopsis PUX proteins. The rice PUX family members were named according to similarity to their Arabidopsis homologs. Numbers next to the branches indicate the percentage of replicated trees in which the associated taxa clustered together in the bootstrap test (1,000 replicates). The scale bar represents the number of amino acid substitutions per site. Domain organization of each protein was visualized using Evolview v3[8]. **b,** Heatmap representation of transcript levels for rice *PUX* genes in different tissues and at different growth stages. Expression data were obtained from the RAP-DP database and visualized using TBtools. **c,** Phylogenetic analysis of OsPUX8B homologs in different plant species. Sequences of OsPUX8B homologs, including different protein versions encoded by different splice variants from *Oryza sativa* (Os), *Medicago truncatula* (Mt), *Brachypodium distachyon* (Bd), *Sorghum bicolor* (Sb), *Zea mays* (Zm)*, Glycine max* (Gm), *Triticum aestivum* (Ta)*, Selaginella moellendorffii* (Sm), *Nicotiana attenuata* (Na), *Arabidopsis thaliana* (At), and *Ostreococcus lucimarinus* (Ol), were obtained from EnsemblPlants. The phylogenetic tree was reconstructed using the same method and parameters as in (**a**). **d,** Domain organization of the three proteins encoded by different splice variants of *OsPUX8B*. UBA, ubiquitin-associated domain; UIM, ubiquitin interaction motif; UBX, ubiquitin regulatory X domain. **e,** Sequence alignment of the UBX domains encoded by the different splice variants of *OsPUX8B* and its homologs. All protein sequence information can be found in Supplementary Data 3.

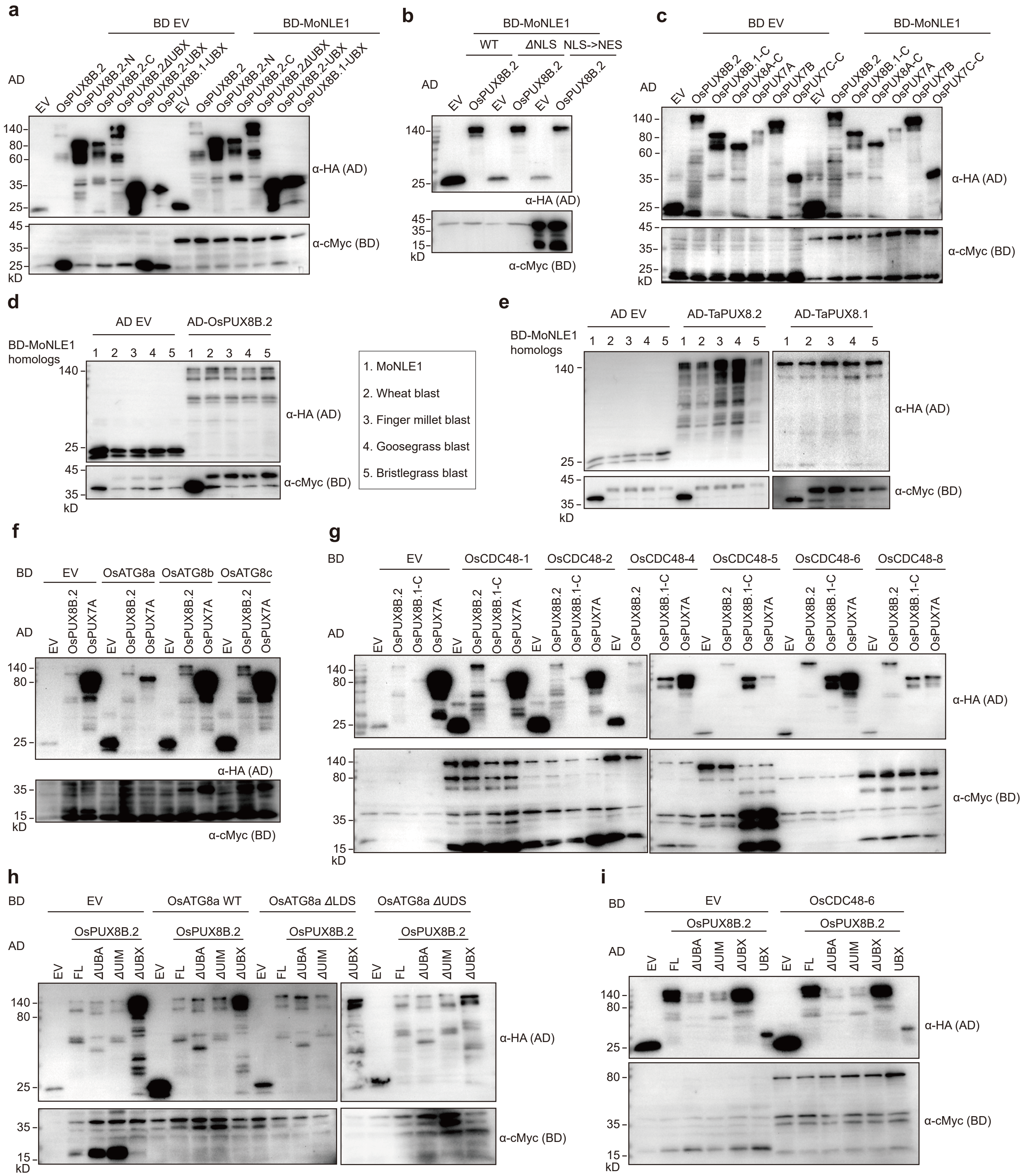

Supplementary Fig. 4 Validation of protein accumulation from Y2H constructs in yeast. a–i, Immunoblot analysis of the indicated proteins in yeast used in Fig. 2c (**a**), Fig. 2d (**b**), Fig. 2e (**c**), Fig 2i (**d**), Fig. 2j (**e**), Fig. 3a (**f**), Fig. 3b (**g**), Fig. 3c (**h**), and Fig. 3f (**i**). The indicated proteins were detected with antibodies recognizing HA and cMyc epitope tags present in the AD and BD constructs, respectively. Data are representatives of three independent experiments with similar results.

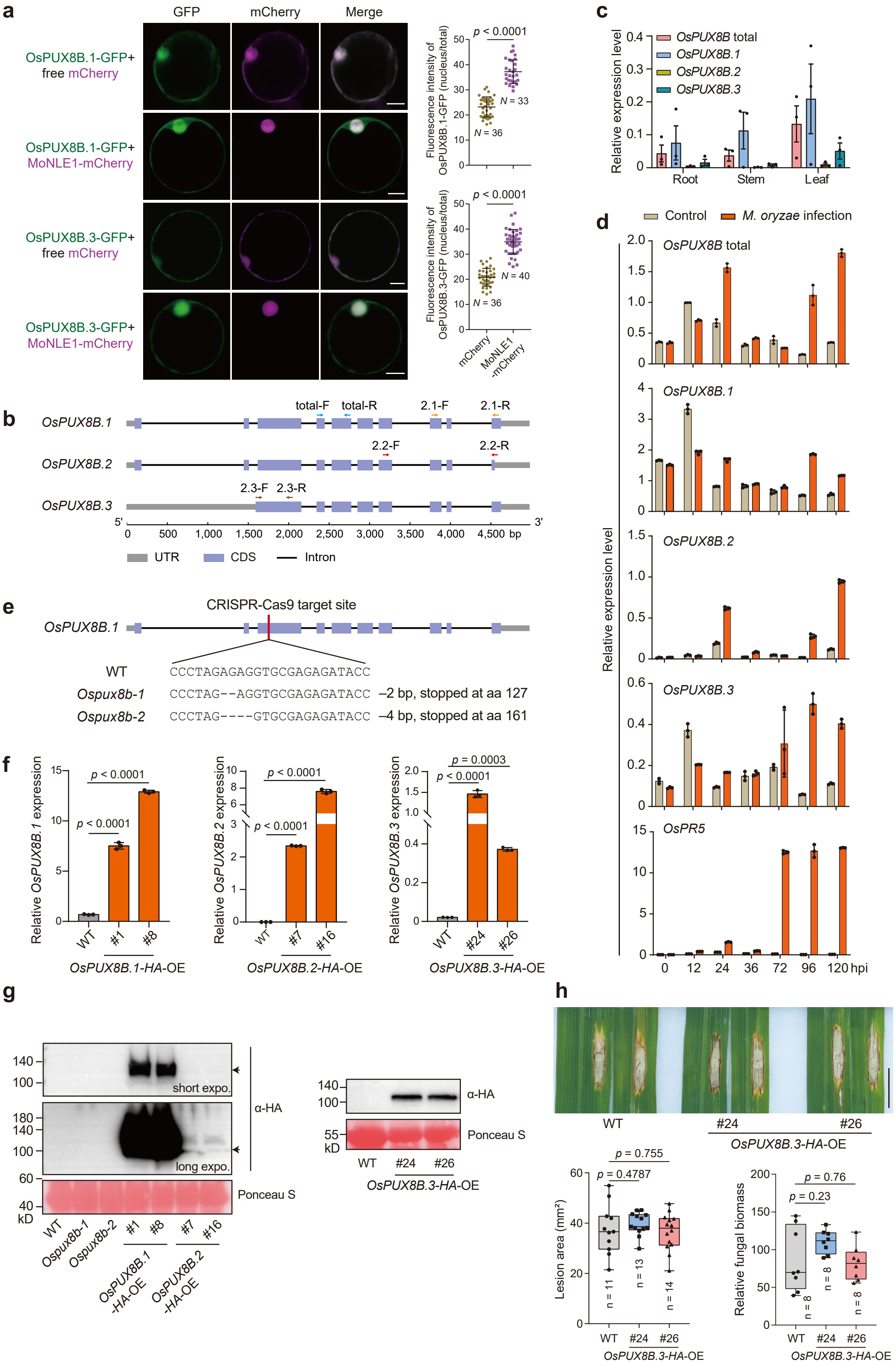

a
GFP
mCherry
Merge
OsPUX8B.1-GFP+ free mCherry
OsPUX8B.1-GFP+ MoNLE1-mCherry
OsPUX8B.3-GFP+ free mCherry
OsPUX8B.3-GFP+ MoNLE1-mCherry
Fluorescence intensity of OsPUX8B.1-GFP (nucleus/total)
Fluorescence intensity of OsPUX8B.3-GFP (nucleus/total)
p < 0.0001
N = 36
N = 33
N = 40
mCherry
MoNLE1 -mCherry
b
OsPUX8B.1
OsPUX8B.2
OsPUX8B.3
total-F
total-R
2.1-F
2.1-R
2.2-F
2.2-R
2.3-F
2.3-R
5'
3'
0
500
1,000
1,500
2,000
2,500
3,000
3,500
4,000
4,500 bp
UTR
CDS
Intron
c
Relative expression level
OsPUX8B total
OsPUX8B.1
OsPUX8B.2
OsPUX8B.3
Root
Stem
Leaf
d
Control
M. oryzae infection
Relative expression level
OsPUX8B total
OsPUX8B.1
OsPUX8B.2
OsPUX8B.3
OsPR5
0
12
24
36
72
96
120 hpi
e
CRISPR-Cas9 target site
OsPUX8B.1
WT CCCTAGAGAGGTGCGAGAGATACC
Ospux8b-1 CCCTAG--AGGTGCGAGAGATACC –2 bp, stopped at aa 127
Ospux8b-2 CCCTAG----GTGCGAGAGATACC –4 bp, stopped at aa 161
f
Relative OsPUX8B.1 expression
Relative OsPUX8B.2 expression
Relative OsPUX8B.3 expression
p < 0.0001
p = 0.0003
WT
#1
#8
#7
#16
#24
#26
OsPUX8B.1-HA-OE
OsPUX8B.2-HA-OE
OsPUX8B.3-HA-OE
g
short expo.
long expo.
α-HA
Ponceau S
kD
WT
Ospux8b-1
Ospux8b-2
#1
#8
#7
#16
OsPUX8B.1 -HA-OE
OsPUX8B.2 -HA-OE
α-HA
Ponceau S
kD
WT
#24
#26
OsPUX8B.3-HA-OE
h
WT
#24
#26
OsPUX8B.3-HA-OE
Lesion area (mm²)
p = 0.755
p = 0.4787
n = 11
n = 13
n = 14
Relative fungal biomass
p = 0.76
p = 0.23
n = 8
WT
#24
#26
OsPUX8B.3-HA-OE

Supplementary Fig. 5 Subcellular localization of OsPUX8B.1 and OsPUX8B.3, structures and expression patterns of different *OsPUX8B* gene models, and generation of *OsPUX8B* knockout/overexpression plants. a, Subcellular localization of OsPUX8B.1 and OsPUX8B.3. *OsPUX8B.1-GFP* or *OsPUX8B.3-GFP* was co-transfected with *mCherry* or *MoNLE1-mCherry* in rice protoplasts. Data were collected at 24 h post transfection (hpt). Ratio (nucleus/total) of fluorescence intensities for OsPUX8B.1-GFP and OsPUX8B.3-GFP when their encoding constructs were co-expressed with *mCherry* or *MoNLE1-mCherry* (*E1-mCherry*) are shown in the dot plots on the right. Data are means ± s.d. from the indicated number of independent biological replicates. **b,** Gene models of *OsPUX8B.1*, *OsPUX8B.2*, and *OsPUX8B.3*. Primers used to identify total or individual transcripts are shown. **c,** Total and individual transcript levels of *OsPUX8B* in roots, stems, and leaves of NPB plants. RT-qPCR was performed using transcript-specific primers shown in (**b**). Data are means ± s.e.m. from three independent biological replicates. **d,** Expression patterns of total *OsPUX8B* and each of the three *OsPUX8B* splice variants upon blast infection. *M. oryzae* isolate RB22 was used to inoculate NPB plants. Data are means ± s.d. from three independent biological replicates. The rice defense marker gene *OsPR5* was used to monitor inoculation efficiency. **e,** Generation of *OsPUX8B* knockout (*ko*) plants in the NPB background. The CRISPR/Cas9 target site in the gene and mutation types of the *ko* mutants are shown. **f, g**, RT-qPCR (**f**) and immunoblot (**g**) analysis of *OsPUX8B.1-HA*-OE, *OsPUX8B.2-HA*-OE, and *OsPUX8B.3-HA*-OE plants in the NPB background. Transcript-specific primers were used for RT-qPCR, with NPB plants used as the wild-type (WT) control. Data are means ± s.d. from three independent biological replicates for RT-qPCR analysis, and data are representatives of three independent experiments with similar results for immunoblot analysis. OsPUX8B.1/B.2/B.3-HA were detected with an anti-HA antibody, and Ponceau S staining was used as a loading control in the immunoblotting assays. **h,** Phenotypes of *OsPUX8B.3-HA*-OE plants against the blast isolate RB22. Lesion area and relative fungal biomass were used to quantify disease symptoms. Scale bar, 1 cm. Data are shown as boxplots displaying the maximum and minimum, first and third quantiles, and the median. n = number of independent biological samples. Data were analyzed by two-tailed unpaired Student's *t*-test (**a**) or one-way ANOVA (**f, h**) followed by Tukey's test. Exact *p* (**a**) or adjusted *p* (**f, h**) values were shown in figures.

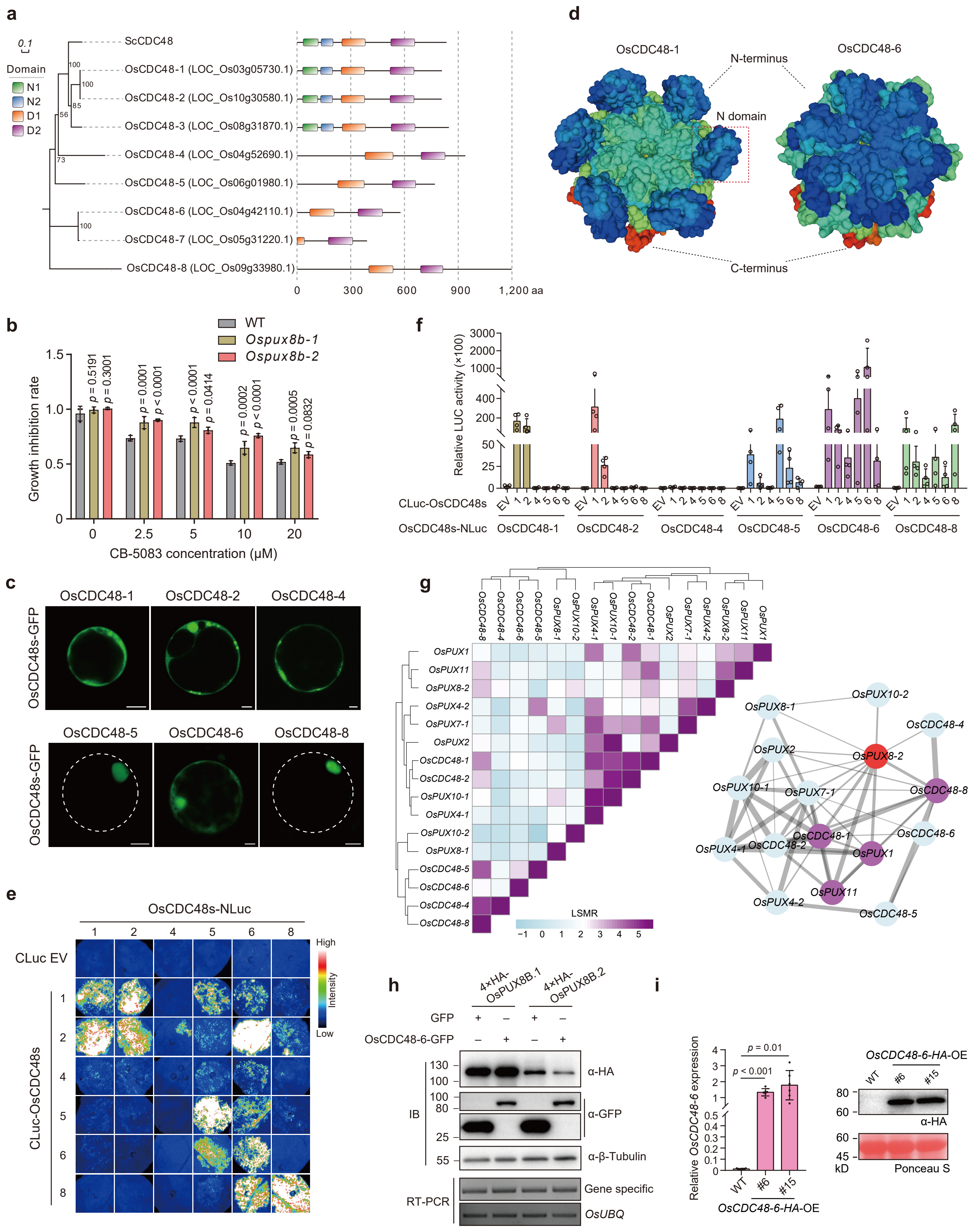

a
Domain
N1
N2
D1
D2
ScCDC48
OsCDC48-1 (LOC_Os03g05730.1)
OsCDC48-2 (LOC_Os10g30580.1)
OsCDC48-3 (LOC_Os08g31870.1)
OsCDC48-4 (LOC_Os04g52690.1)
OsCDC48-5 (LOC_Os06g01980.1)
OsCDC48-6 (LOC_Os04g42110.1)
OsCDC48-7 (LOC_Os05g31220.1)
OsCDC48-8 (LOC_Os09g33980.1)
0 300 600 900 1,200 aa
d
OsCDC48-1
OsCDC48-6
N-terminus
N domain
C-terminus
b
WT
Ospux8b-1
Ospux8b-2
Growth inhibition rate
CB-5083 concentration (μM)
c
OsCDC48s-GFP
OsCDC48-1 OsCDC48-2 OsCDC48-4
OsCDC48-5 OsCDC48-6 OsCDC48-8
e
OsCDC48s-NLuc
CLuc EV
CLuc-OsCDC48s
High
Intensity
Low
f
Relative LUC activity (×100)
CLuc-OsCDC48s
OsCDC48s-NLuc
OsCDC48-1 OsCDC48-2 OsCDC48-4 OsCDC48-5 OsCDC48-6 OsCDC48-8
g
LSMR
h
4×HA-OsPUX8B.1
4×HA-OsPUX8B.2
GFP
OsCDC48-6-GFP
IB
α-HA
α-GFP
α-β-Tubulin
RT-PCR
Gene specific
OsUBQ
i
Relative OsCDC48-6 expression
OsCDC48-6-HA-OE
α-HA
Ponceau S
kD

Supplementary Fig. 6 Characterization of rice CDC48 proteins. a, Phylogenetic analysis of rice CDC48 proteins. Sequences for rice CDC48 proteins were obtained using a BLASTP search (similarity > 50%, *e*-value < 10^{-5}) against the rice protein database using yeast (*Saccharomyces cerevisiae*) CDC48 as a query, based on their high sequence conservation in eukaryotes. The tree was reconstructed using similar parameters used in Supplementary Fig. 3a. The right panel shows the domain distributions in these proteins. **b,** Responses of *Ospux8b* mutants to CB-5083 treatment. Growth inhibition rates were calculated by comparing the fresh weights of plants representing each line treated with the indicated concentration of CB-5083 with those of untreated plants. Data are means ± s.d. from three independent biological replicates. The adjusted *p* values were determined by two-way ANOVA followed by Tukey's test. **c,** Subcellular localization of rice CDC48 proteins. *35S:OsCDC48s-GFP* constructs were transfected into rice protoplasts individually, and fluorescence signals were observed at 24 hpt. Dotted lines show cell outlines, and arrowheads indicate nuclei. Data are representatives of three independent experiments with similar results. **d,** Homology modeling of OsCDC48-1 and OsCDC48-6. The hexameric structures of OsCDC48-1 and OsCDC48-6 were built in SWISS-MODEL by homology modeling according to the human p97 structure (PDB: 7LMY). **e, f** Split-luciferase complementation (SLC) assay examining the associations between rice CDC48 proteins. Representative luminescence images (**e**) and relative luciferase activity (**f**) were recorded at 48 h post Agrobacterium infiltration. Data in (**f**) are means ± s.d. from four independent biological replicates. **g,** Co-expression analysis of rice *PUX* and *CDC48* genes. The logistic mutual ranks (LSMRs) for the corresponding pairs of genes were obtained from ATTED-II and are displayed as a correlation matrix. A co-expression network for *OsPUX8B* was constructed using Cytoscape with edge weights representing LSMRs. **h,** OsCDC48-6 promotes OsPUX8B.2 degradation but does not affect OsPUX8B.1 stability. Similar results were observed in three independent experiments. **i,** RT-qPCR (left panel) or immunoblot (right panel) analysis to identify *OsCDC48-6-HA*-OE plants in the NPB background. Data are means ± s.d. from six independent biological replicates for RT-qPCR. The adjusted *p* values were determined by one-way ANOVA followed by Tukey's test. OsCDC48-HA was detected with an anti-HA antibody, and Ponceau S staining was used as loading control in the immunoblotting assay. Data are representatives of three independent experiments with similar results for immunoblot analysis.

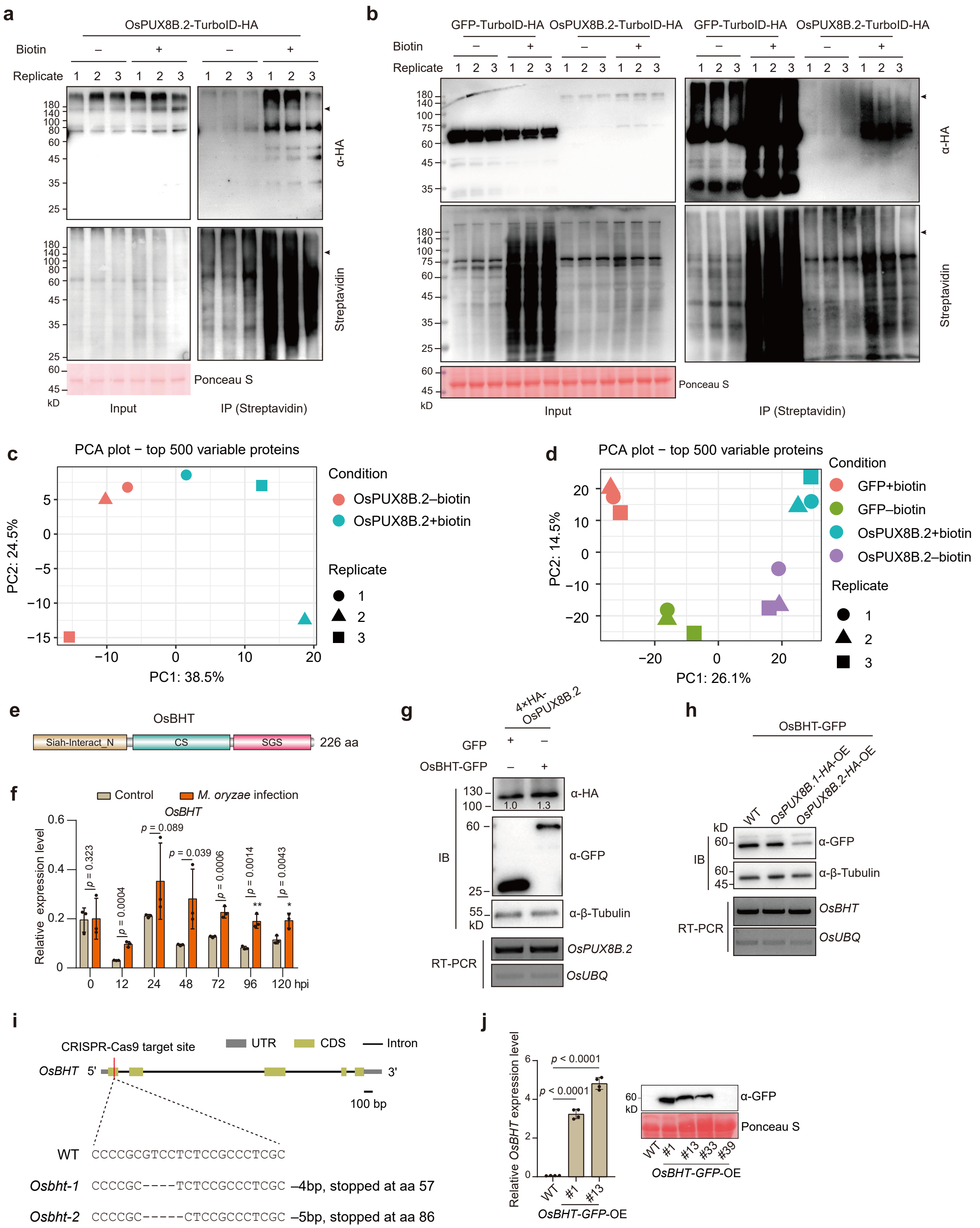

a
OsPUX8B.2-TurboID-HA
Biotin
Replicate
α-HA
Streptavidin
Ponceau S
kD
Input
IP (Streptavidin)
b
GFP-TurboID-HA
OsPUX8B.2-TurboID-HA
c
PCA plot − top 500 variable proteins
PC1: 38.5%
PC2: 24.5%
Condition
OsPUX8B.2–biotin
OsPUX8B.2+biotin
Replicate
d
PCA plot − top 500 variable proteins
PC1: 26.1%
PC2: 14.5%
GFP+biotin
GFP–biotin
e
OsBHT
Siah-Interact_N
CS
SGS
226 aa
f
Control
M. oryzae infection
OsBHT
Relative expression level
hpi
g
4×HA-OsPUX8B.2
GFP
OsBHT-GFP
IB
α-GFP
α-β-Tubulin
RT-PCR
OsPUX8B.2
OsUBQ
h
WT
OsPUX8B.1-HA-OE
OsPUX8B.2-HA-OE
OsBHT
i
CRISPR-Cas9 target site
UTR
CDS
Intron
100 bp
WT CCCCGCGTCCTCTCCGCCCTCGC
Osbht-1 CCCCGC----TCTCCGCCCTCGC –4bp, stopped at aa 57
Osbht-2 CCCCGC-----CTCCGCCCTCGC –5bp, stopped at aa 86
j
Relative OsBHT expression level
p < 0.0001
OsBHT-GFP-OE

Supplementary Fig. 7 Identification of OsPUX8B.2-interacting proteins using the proximity-labeling method. a, b Immunoblot verification of biotin-labeled proteins before mass spectrometry analysis. The input and immunoprecipitated protein samples without (**a**) or with (**b**) MG132 treatment (three biological replicates for each treatment) were detected with anti-HA and streptavidin-conjugated antibodies in the immunoblot analysis. Arrowheads indicate the expected sizes of OsPUX8B.2-TurboID-HA. Ponceau S staining was used as an input loading control. **c, d**, Principal component analysis (PCA) of the proximity-labeling spectrometry data for MG132-untreated (**c**) or MG132-treated samples (**d**). **e,** Domain organization of OsBHT. Siah-Interact_N, Siah interacting protein, N terminal; CS, CHORD (cysteine- and histidine-rich domain)-containing protein and SGT1 domain; SGS, SGT1-specific domain. **f,** Expression pattern of *OsBHT* upon rice blast (strain RB22) infection. Data are means ± s.d. from three independent biological replicates. The exact *p* values were determined by multiple two-tailed unpaired Student's *t*-tests. **g,** Effect of OsBHT on OsPUX8B.2 stability. Numbers below the protein bands indicate relative OsPUX8B.2-HA levels compared to β-tubulin. **h,** OsBHT is more unstable in protoplasts prepared from *OsPUX8B.2-HA*-OE plants than from wild-type (WT) plants. Equivalent amounts of *OsBHT-GFP* plasmid DNA were transfected into protoplasts prepared from the indicated plants. At 24 hpt, protoplasts were treated with 100 μM cycloheximide (CHX) for 10 h followed by collection and immunoblotting. **i,** Validation of *Osbht* mutant plants. The CRISPR/Cas9 target site in the *OsBHT* genome sequence and the mutation type for each *ko* mutant line are shown. **j,** RT-qPCR (left panel) or immunoblot (right panel) analysis of *OsBHT-GFP*-OE plants. Data are means ± s.d. from four independent biological replicates for RT-qPCR. The adjusted *p* values were determined by one-way ANOVA followed by Tukey's test. OsBHT-GFP was detected with an anti-GFP antibody, and Ponceau S staining was used as loading control in the immunoblotting assay. Immunoblot results in (**g**, **h** and **j**) are representatives of three independent experiments with similar results.

Supplementary references:

1. Kim, S. et al. Two nuclear effectors of the rice blast fungus modulate host immunity via transcriptional reprogramming. *Nat Commun* 11, 5845, doi:10.1038/s41467-020-19624-w (2020).
2. Almagro Armenteros, J. J. et al. SignalP 5.0 improves signal peptide predictions using deep neural networks. *Nat Biotechnol* 37, 420-423, doi:10.1038/s41587-019-0036-z (2019).
3. Sperschneider, J., Dodds, P. N., Singh, K. B. & Taylor, J. M. ApoplastP: prediction of effectors and plant proteins in the apoplast using machine learning. *New Phytol* 217, 1764-1778, doi:10.1111/nph.14946 (2018).
4. Kall, L., Krogh, A. & Sonnhammer, E. L. A combined transmembrane topology and signal peptide prediction method. *Journal of molecular biology* 338, 1027-1036, doi:10.1016/j.jmb.2004.03.016 (2004).
5. Almagro Armenteros, J. J. et al. Detecting sequence signals in targeting peptides using deep learning. *Life Sci Alliance* 2, doi:10.26508/lsa.201900429 (2019).
6. Krogh, A., Larsson, B., von Heijne, G. & Sonnhammer, E. L. Predicting transmembrane protein topology with a hidden Markov model: application to complete genomes. *Journal of molecular biology* 305, 567-580, doi:10.1006/jmbi.2000.4315 (2001).
7. Sperschneider, J. & Dodds, P. N. EffectorP 3.0: Prediction of Apoplastic and Cytoplasmic Effectors in Fungi and Oomycetes. *Mol Plant Microbe Interact* 35, 146-156, doi:10.1094/MPMI-08-21-0201-R (2022).
8. Subramanian, B., Gao, S., Lercher, M. J., Hu, S. & Chen, W. H. Evolview v3: a webserver for visualization, annotation, and management of phylogenetic trees. *Nucleic Acids Res* 47, W270-W275, doi:10.1093/nar/gkz357 (2019).
