# Supplementary material for: A fungal core effector exploits the OsPUX8B.2–OsCDC48-6 module to suppress plant immunity
Source: Nat Commun. 2024 Mar 22;15:2559. doi: 10.1038/s41467-024-46903-7 (PMC10959940; doi:10.1038/s41467-024-46903-7)
Supplement: Supplementary file 3 — Description of Additional Supplementary Files [file 41467_2024_46903_MOESM3_ESM.pdf]

Description of Additional Supplementary Files:

Supplementary Data 1: Predicted cNLS-harboring proteins secreted by Magnaporthe oryzae during infection.

Supplementary Data 2: A diverse collection of 187 M. oryzae isolates from 13 distinct host genera obtained from previous publications.

Supplementary Data 3: Information about the proteins used in this study.

Supplementary Data 4: Proteins probed by OsPUX8B.2-TurboID-HA using proximitylabeling spectrometry.

Supplementary Data 5: Primers used in this study.
